# Complete Genome Sequence of *Streptomyces lavendulae* subsp. *lavendulae* CCM 3239 (Formerly “*Streptomyces aureofaciens* CCM 3239”), a Producer of the Angucycline-Type Antibiotic Auricin

**DOI:** 10.1128/genomeA.00103-18

**Published:** 2018-03-01

**Authors:** Tobias Busche, Renata Novakova, Arwa Al'Dilaimi, Dagmar Homerova, Lubomira Feckova, Bronislava Rezuchova, Erik Mingyar, Dominika Csolleiova, Carmen Bekeova, Anika Winkler, Beatrica Sevcikova, Jörn Kalinowski, Jan Kormanec, Christian Rückert

**Affiliations:** a Center for Biotechnology (CeBiTec), Universität Bielefeld, Bielefeld, Germany; b Institute of Molecular Biology, Slovak Academy of Sciences, Bratislava, Slovakia

## Abstract

Streptomyces lavendulae subsp. lavendulae CCM 3239 produces the angucycline antibiotic auricin and was thought to be the type strain of Streptomyces aureofaciens. We report the complete genome sequence of this strain, which consists of a linear chromosome and the linear plasmid pSA3239, and demonstrate it to be S. lavendulae subsp. lavendulae.

## GENOME ANNOUNCEMENT

The Gram-positive soil bacteria of the genus Streptomyces undergo exceptional morphological differentiation, accompanied by “physiological differentiation,” characterized by production of biologically active secondary metabolites like antibiotics (1). The strain “Streptomyces aureofaciens CCM 3239” (where the quotes indicate that the strain was originally misidentified as S. aureofaciens), received from the Czech Collection of Microorganisms (CCM), has been investigated for more than 25 years by our group with regard to morphological differentiation and the role of sigma factors therein (2–9).

We also identified the type II polyketide gene cluster *aur1*, located on the linear plasmid pSA3239. This gene cluster encodes production of the unique angucycline antibiotic auricin, which is produced during a narrow interval of several hours after entry into stationary phase, after which it is degraded due to its instability at high pH values reached later in the stationary phase. This unusual pattern of auricin production arises from its strict complex regulation, involving both feed-forward and feedback control by auricin intermediates via several transcriptional regulators (9–12). Thus, the genome sequence of “S. aureofaciens CCM 3239” should aid in the understanding of differentiation and global regulatory mechanisms for auricin biosynthesis in this strain.

Purified genomic DNA (gDNA) of “S. aureofaciens CCM 3239” was isolated with standard methods (13). Sequencing, assembly, and finishing were done as described previously (14) using a TrueSeq DNA PCR-free library instead of a Nextera WGS library. Assembly of 1.5 Gbp raw data resulted in 4 scaffolds containing 181 contigs. Gene prediction and annotation were performed using Prokka (15). The linear chromosome has a size of 8,691,831 bp (72.63% G+C content), while the linear plasmid pSA3239 has a size of 241,081 bp (72.12% G+C). Automated annotation of the complete genome sequence revealed the presence of 8,101 genes, including 159 RNA-encoding genes. Analysis of the genome sequence with the tool antiSMASH (16) revealed the presence of 26 respectively 5 putative secondary metabolite gene clusters located on the chromosome respectively pSA3239.

Intriguingly, comparison of the 16S rRNA of “S. aureofaciens CCM 3239” with that of the type strain S. aureofaciens NBRC 12594 (17) revealed a low similarity (93% identity). Analysis of the 16S rRNA sequence in the ribosomal database project (18) using type strains revealed the highest similarity (99.3%) to Streptomyces lavendulae IFO 12789. Moreover, the genome sequence of “S. aureofaciens CCM 3239” was 100% identical with contigs of the draft sequence of Streptomyces lavendulae subsp. lavendulae NRRL B-2774 (GenBank accession number NZ_JOEW00000000), including the 16S rRNA genes. In addition, the genome sequence contains a gene cluster for the synthesis of the antibiotic streptothricin, which is identical on the nucleotide level to the gene cluster from Streptomyces lavendulae subsp. lavendulae NBRC 12789 (19). The lavender spore color and the production of soluble brown pigments provide additional phenotypic evidence that strain CCM 3239 is wrongly filed by CCM and is actually S. lavendulae subsp. lavendulae CCM 3239.

### Accession number(s).

The complete genome sequence was deposited at DDBJ/ENA/GenBank under the accessions numbers CP024985 (chromosome) and CP024986 (plasmid).
